# Supplementary material for: From sequence to enzyme mechanism using multi-label machine learning
Source: BMC Bioinformatics. 2014 May 19;15:150. doi: 10.1186/1471-2105-15-150 (PMC4229970; doi:10.1186/1471-2105-15-150)
Supplement: Additional file 2 — Java code of ml2db. Additional file ml2db_code.tar.gz contains the Java source code to run the multi-label machine learning experiments and save the results to database. The code’s Javadoc is included. [file 1471-2105-15-150-S2.zip › additional file 2/ml2db/ml2db/doc/mulan/classifier/meta/thresholding/package-summary.html]

mulan.classifier.meta.thresholding


JavaScript is disabled on your browser.


- Overview
- Package
- Class
- Use
- Tree
- Deprecated
- Index
- Help

- Prev Package
- Next Package

- Frames
- No Frames

- All Classes

# Package mulan.classifier.meta.thresholding

- Class Summary

  | Class | Description |
  | --- | --- |
  |  |  |
  | --- | --- |
  | ExampleBasedFMeasureOptimizer | This class takes the marginal probabilities estimated for each label by a multi-label learner and transforms them into a bipartition which is approximately optimal for example-based FMeasure. |
  | Meta | Base class for instance-based prediction of a bipartition from the labels' scores |
  | MetaLabeler | Class implementing the MetaLabeler algorithm. |
  | MLPTO | Class that implements the Multi Label Probabilistic Threshold Optimizer (MLTPTO). |
  | OneThreshold | Class that estimates a single threshold for all labels and examples. |
  | RCut | Classs that implements RCut(Rank-based cut). |
  | SCut | Class that implements the SCut method (Score-based local optimization). |
  | ThresholdPrediction | Class that learns to predict a different threshold per exampleFor more information, see    Elisseeff, Andre, Weston, Jason: A kernel method for multi-labelled classification. |

- Overview
- Package
- Class
- Use
- Tree
- Deprecated
- Index
- Help

- Prev Package
- Next Package

- Frames
- No Frames

- All Classes
